# Editorial: RNA-Mediated Processes in Epigenetics; an Integrative View in the Maintenance of Homeostasis

**DOI:** 10.3389/fgene.2020.629918

**Published:** 2021-02-03

**Authors:** Bertrand Kaeffer, Hanna Taipaleenmäki, Sandra L. de Souza

**Affiliations:** ^1^Nantes University, INRAE, UMR 1280, PhAN, Nantes, France; ^2^Molecular Skeletal Biology Laboratory, Department of Trauma, Hand, and Reconstructive Surgery, University Medical Center Hamburg-Eppendor, Hamburg, Germany; ^3^Laboratory of Neuroplasticity and Behavior, Department of Anatomy, Federal University of Pernambuco, Recife, Brazil

**Keywords:** circular RNA, early life stress, obesity, breast milk, social isolation, non-coding RNAs, extracellular vehicles, cardiovascular disease(s)

Epigenetics is a mechanism linking environmental factors to altered gene activity that are associated with environmental factors, and includes mechanisms such as DNA methylation, histone modifications and RNA-mediated processes. Epigenetic changes can hold the memory of the effects of environmental factors to which an individual is subjected throughout his life. These mechanisms are implicated in the selection of new phenotypes and proposed to reassess the theory of natural selection (Guerrero-Bosagna, 2017). Non-coding RiboNucleic Acids (small or long ncRNA) are involved in epigenetic regulation directly silencing or activating chromatin at specific loci or through their integral role in the machinery that drives DNA methylation. The Research Topic incorporates new data on the epigenetic effects of non-coding RNAs on generation F0, then presents the transgenerational influences of non-coding RNAs on the F1 and F2 generations.

The first group of papers explores the short-term epigenetic effects on the F0 generation detailing the molecular events related to the regulation by miRNAs and long ncRNAs.

In the bioprocessing of true miRNAs like miR-21, both the 3p and 5p molecules are expressed, each with a specific functional effect in various cells (Desvignes et al., 2015; Alles et al., 2019). Dai et al. describe the cell-type-specific functions of miR-21 in different cardiovascular diseases and its potential use in clinical therapy. The incidence of miRNA-21-5p or−3p is clearly shown opening an original perspective on the biological meaning of 3p and 5p importance in miRNA evolution (The 5p strand is present in the forward (5′-3′) position, while the 3p strand is located in the reverse position of the pre-miRNA hairpin). The paper is also rightfully pointing to the limitation of algorithms in the prediction of binding sites of miR-21 on PTEN, underlining the need for biological validation for predicted targets.

In their study of the intestine, Ruiz-Roso et al. use skillfully-designed experiments on Dicer-1 knock-out mouse and human organoids to investigate the expression of miRNA-regulated cholesterol and lipoprotein metabolism-related genes and proteins involved in the homeostatic regulatory machinery of the postprandial lipemia. In doing so, they are identifying potential novel therapeutic targets in lipid metabolism disorders. Outside the field of human diseases, Wang et al. provide a detailed insight into how miR-149-5p plays a crucial role in agronomic products derived from domestic mammals. The implication of a specific miRNA in the production of top-quality hair is not only of interest in the textile industry but also in the preservation of highly valuable domestic mammal races. Lin et al. discuss the properties of long ncRNAs as a biomarker of Tumor MicroEnvironment in bladder cancer and their links with miRNA. They emphasize cogently the importance of examining the levels of infiltrated immune cells in the tumor (T cells or myeloid cells). Indeed, the immune response is crucial in regard to proper cellular communication. Bosch et al. 's pioneering opinion paper has a similar focus. The TRL-7/8 toll-like receptors bind miRNAs and they are expressed on endosome membranes, suggesting that TLR-binding microRNAs transported via extracellular vesicles probably serve in stress responses.

The cellular microenvironment is also especially relevant to the growth of bone during tumorigenesis (Haider and Taipaleenmäki, 2018) and in the epigenetic regulation of skeletal impairment due to a high-fat diet (Tencerova et al., 2018). Penolazzi et al. have discussed the importance of joint homeostasis in skeletal development and are persuasive in their call for an international consortium on 3D models.

The second group of papers is exploring the long-term epigenetic effects on F1 and F2 generations.

In the literature, Harman et al. (2020) are proposing the first demonstration documenting the reprogramming of heritability in mammals to promote disease resilience in the next generation. A beneficial link of stress on the F1 generation connected with some miRNA pathway has been described on a mouse model of ophthalmic care. In nutrition, the microRNAs are now on the way to becoming new micronutrients according to Wang et al. (2018) who demonstrate the passage of microRNAs from cow's milk into consumer's plasma.

In this Research Topic, Ozkan et al. have cleverly designed a mouse model on breast milk siblings. The findings support the theory, for the first time, that the factors modifying the epigenetic mechanisms may be transmitted by breast milk and these epigenetic interactions may be transferred to offspring. These results are also suggesting hereditary epigenetic effects of cross-fostering on future generations and the impact of mother-infant dyad on epigenetic programming through miRNAs. Such works are calling for a future reappraisal of milk banking practices, but also on the conception of supplementation distributed during breast-feeding (Moro and Arslanoglu, 2020).

In addition to the stress of being breast-fed by an adoptive mother, there are others that can have deleterious consequences on adult life. Tavares et al. have identified an interesting relationship between Early Life Stress and the modulation of the serotonergic and dopaminergic systems, through post-transcriptional regulation by miRNAs. The use of miRNAs supplementation to prevent mid-term consequences of early weaning stress is under study with miR-320-3p (Tavares et al., 2020), a non-canonical miRNA with a non-described 5p form (Desvignes et al., 2015). Arzate-Mejía et al. provide a comprehensive overview of the effects of prolonged periods of social isolation with a focus on the molecular events leading to behavioral alterations (related to memory or cognition but also relevant for the modulation of mood and even of addictive behaviors). Important epigenetic modifiers such as the H3K9me2 histone methyltransferase G9a and histone deacetylases like HDAC-2 and−4, as well as regulatory ncRNAs like microRNAs, are also dysregulated, suggesting that social isolation could remodel chromatin and impact steady-state or stimulus-dependent transcriptional responses.

In conclusion, this eBook illustrates the complexity of epigenetic regulation through the valuable contributions of the authors, and is pioneering new avenues in the molecular regulation of homeostasis, diabetes, obesity and cardiovascular and psychiatric diseases related to environmental stress.

## Author Contributions

All authors have contributed and validated the editorial.

## Conflict of Interest

The authors declare that the research was conducted in the absence of any commercial or financial relationships that could be construed as a potential conflict of interest.
